# Hospital-borne hazardous air pollutants and air cleaning strategies amid the surge of SARS-CoV-2 new variants

**DOI:** 10.1016/j.heliyon.2024.e38874

**Published:** 2024-10-05

**Authors:** Nishant Gupta, N.S. Abd EL-Gawaad, L.O. Mallasiy

**Affiliations:** a Medical Research & Development, River Engineering Private Limited, Ecotec-3, Greater Noida, India; b Department of Physics, Faculty of Science, King Khalid University, Abha, 62529, Saudi Arabia; c Department of Home Economics, Faculty of Science and Arts in Tihama, King Khalid University, Muhayil Asir, 61913, Saudi Arabia

**Keywords:** Indoor air pollutants, High ventilation, Wards design, Passive removal materials, Air purifier, Advance HVAC, Bipolar air ionization, Ultraviolet C, Trombe wall

## Abstract

Indoor air pollutants and airborne contamination removal have been challenging in healthcare facilities. The airborne transmission control and HVAC system may collapse in hospitals due to the highly infectious respiratory disease-associated patient surge, like COVID-19. Common air filtration systems and HVAC systems enhance the patients' comfort and support indoor hygiene, hitherto insufficient to control highly infectious airborne pathogens and hospital-borne pollutants such as radon, PM_2.5_, patient droplets, VOC, high CO_2_, and anesthetic gases. This review summarized important air cleaning interventions to enhance HVAC efficiency and indoor safety. We discussed efficient air cleaning and ventilation strategies including air filtration, air ionization, passive removal materials (PRM), and UVGI to minimize cross-contamination in hospital wards.

## Graphical abstract

**Figure:**
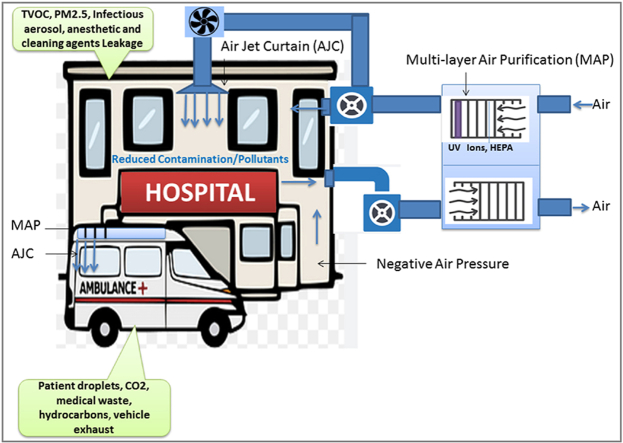

## 1 Introduction

Indoor air quality and associated health risks have been one of the critical subjects during the COVID-19 pandemic. Areas such as surgical theaters, ICU, and isolation wards are susceptible to multiple airborne contaminants and pollutants due to continuous exposure of respiratory droplets, skin squames, lint, respiratory droplets, aerosols, disinfection and sterilizing substances, outdoor pollutants, and leakage of anesthetic gasses [1]. Hospital-acquired infections represent serious health burdens and mortality worldwide [2,3,4,5].

Indoor air pollutants may aggravate and facilitate respiratory infection spread, particulate pollutants particles may provide substrate to airborne pathogens deposition and transmission [6,7].

According to the World Health Organization (WHO), indoor air pollution causes 3.8 million deaths annually. Indoor air pollution short- and long-term exposure may cause a wide range of diseases.In 2020, COVID-19 transmission was highest in healthcare facilities. However, healthcare-associated airborne and other infections and antimicrobial resistance-associated worldwide mortality are uncountable. It is observed that around 24 % of patients are affected by healthcare-associated sepsis and 52.3 % of those patients die each year when treated in an intensive care unit. In the case of antimicrobials resistant deaths are increased by two to threefold [8,[9], [10],^11^].

Besides airborne infections, cleaning agents and detergents may deteriorate indoor air quality as raise indoor HCHO and Volatile Organic Compounds (VOCs) concentrations in hospital environments. PM_2.5_ concentration in the patients' wards may reach more than outdoors in polluted regions. Increased exposure to VOC and particulate pollutants may come with several negative effects on patients and hospital staff [12,13]. Disinfectants, sanitizers, medical equipment, polymer materials-based blood bags, injectors plastic, infusion bags plastic film, and rubber tubing may be a potential source of phthalate and VOC. Air pollutants radon, anesthetic gases such as halothane, isoflurane, sevoflurane, and N_2_O may also occur in hospitals [[14], [15]]. Microbial count may reach up to 10^4^ cells m^3^ in hospital air. So pollutant-free air supply could be challenging in hospital settings [16]. A recent study in India indicates that outside and inside air pollutant types and concentrations are influenced by the surrounding area where hospitals are situated, for instance particulate matter M_1.0–2.5_, PM_0.50–1.0_, PM_0.25–0.50_, PM _< 0.25_, and indoor fungi load are highest in the industrial belt. In contrast, PM _> 2.5_ and bacterial load are higher in residential belts [17]. Similarly, indoor air pollutants negative impact and hospital-acquired infections (HAI) is 19 % higher in developing countries due to high air pollution. HAI-associated financial burden due to antimicrobial resistance bacteria such as Methicillin-resistant *Staphylococcus aureus* (MRSA), and carbapenem-resistant *Enterobacterales* is more in polluted regions [18,19]. Air disinfection is a routine and integral part of hospital cleaning. Air Handling Unit (AHU) systems Including Heating, ventilation, and air-conditioning (HVAC) are crucial in providing a compatible safe environment for hospital patients, filtering dust particles, and biological contaminants, and maintaining air change rates and ventilations. Nevertheless, the chance of air-conditioning-associated microbial transmission remains uncertain [20,21].

Hitherto, the risk of secondary transmission of contaminants in healthcare facilities is high as many hospitals' capacity is limited to safely handle the surge of infectious patients [22]. Sneezing, coughing, biological waste products, spraying of liquids, and dust generate aerosolized particles. Aerosolized particles that are smaller than 100 μm float and are dispersed via air currents to different places [23]. Air pollutants CO_2_, PM_2.5_, PM_10_, TVOCs, patient droplets, and anesthetic gasses leakage remain active and entrapped due to the packed indoor atmosphere and affect hospitals and ambulance services [24,25]. Indoor ventilation design and air cleaning are crucial in healthcare facilities since any glitch in the air-conditioning system may lead to serious consequences [26,27]. Air conditioning units themselves may also act as reservoirs of contaminants. A study in 25 operating theatres in India showed around 26 % of AC units filter with fungal colonization [28].

An obstructed airflow or poor HVAC maintenance can increase the pathogen concentrations in healthcare facilities. There are limited studies on indoor air quality management in health sectors to tackle the sudden surge of highly infectious patients. Few studies suggest that good ventilation with fibrous filters (HEPA), ultraviolet radiation, or plasma units may reduce the biological droplet and chemical contaminants [29,30,31].

Considering this context, this review discussed how the healthcare air conditioning/filtration system can be improved with advanced air filtration, disinfection, and ventilation techniques. Control of hospital-borne aerosol reduces the chances of contaminants exposure and enhances the safety preparedness against the potential risk of nosocomial outbreaks, occupational airborne pollutants, and COVID-19-like highly contagious transmission.

## 2 Methodology

**Fig. 1:**
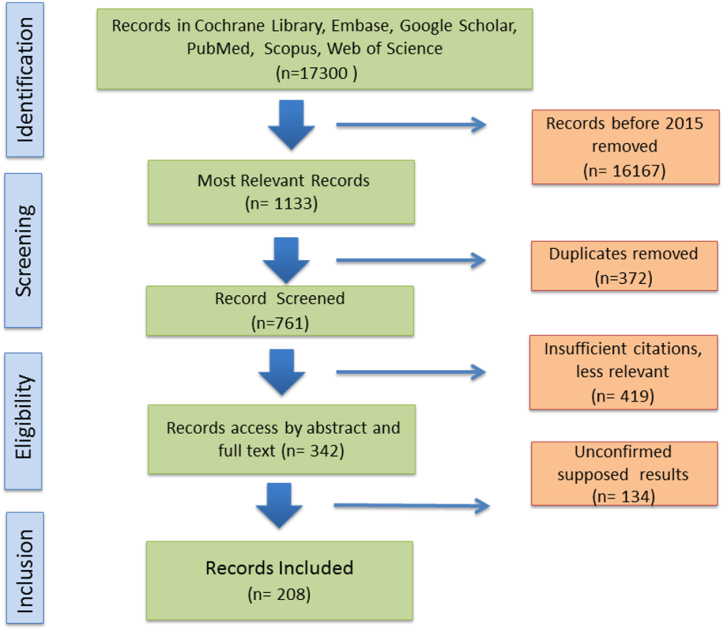
Schematic representation of methodology.

This review is comprised of recent literature related to the theme. A total of 17300 relevant records were found in PubMed, Scopus, Web of Science, Embase, Cochrane Library, and Google Scholar. Searching filters such as “Air Pollutants in Hospitals” “HVAC in hospitals” and “air cleaning in hospitals” from 2015 to 2023 time period were used during the draft preparation and writing, and more keywords were used during revision to fill the gaps suggested by reviewers. Articles selection criteria were based on the number of citations. Irrelevant/duplicate articles were removed, and 188 articles were incorporated into the final version of the paper, Fig. 1.

Healthcare-associated contaminants and their possible removal strategies are summarized under the following main 7 sections, numbered as.

3. Potentially hazardous air pollutants in healthcare facilities.
4. Airborne droplets Transmission Control in different Wards
5. Air Filtration Strategies
6. Advanced HVAC System
7. Photocatalytic oxidation (PCO), plants and Trombe wall role to manage nearby outdoor air pollutants
8. Organic pollutants abatement by Passive Removal Materials (PRMs)
9. Air cleaning Intervention in Emergency Health Vehicles

## 3 Potentially hazardous air pollutants in healthcare facilities

Hospital wards, especially surgical theatres are among the most demanding healthcare work areas with the potential exposure of contaminated air containing disinfection and sanitizing substances, and anesthesia gasses Fig. 4. The concentration of CO_2_, VOCs, and PM_2.5_ may increase in clinics. Therefore, HVAC installations with air-cleaning interventions are recommended to provide a safe environment for surgeons, medical staff, and patients [32,1]. Poor air quality can interfere with patients’ recovery and also cause sudden outbreaks of sick-building syndrome including nausea, fatigue, headaches, and eye irritation. Moreover, the risk of high indoor air pollutants could be immensely high in most polluted countries due to the positive correlation between outdoor and indoor PM_1.0_ and PM_2.5_ concentrations [33,34]. Indoor air pollutants in hospital environments may cause several negative health conditions, Table 1. A study in China indicated that total volatile organic compounds (TVOC) such as formaldehyde, toluene, and xylene, etc. are the main carcinogenic indoor air pollutants [35,36].

**Table 1:** Hospital-borne air contaminants associated health complications and their removal techniques.

Indoor Air Pollutants and contaminants	Potential Exposure Source in Hospitals	Health Impact	Source Control & Cleaning Techniques
Asbestos (Mg_3_(Si2O_5_)(OH)_4_)	Asbestos-containing building materials in surfacing materials, thermal system insulation [37]	Lung cancer (asbestosis) and mesothelioma [[38], [39], [40]]	Water misting [41]
Arsenic (As)	Nearby industries, orchards, coal combustion, pesticide residues [42]	Extremely toxic, lung damage, shortness of breath, chest pain, and cough. Long term Exposure can lead the development of various cancer [43,44]	Several types of adsorbents such as Calcium-based adsorbents and ion exchanges [45,46]
Biological Pollutants (Pollen, viruses, bacteria, mold, dried allergens)	Mostly operating rooms and intensive care units [47]	Infectious illnesses, hypersensitivity, allergies, dizziness [39,43]	High ventilation [48], downward ventilation [49], HEPA Filters, portable air cleaners (if HVAC not functional), negative-pressure, Recommended Ventilation 80–160 L/s/patient for airborne isolation wards 60 L/s/patient for general wards 2.5/sec/m^3^ for corridors [50] Or 6-12 ACH for new building and about 6 ACH in existing buildings [50]
TVOC (Benzene, HCHO)	Anesthetic gases, disinfectant, hand sanitiser, pharmaceuticals and cleaning products [51] Pathology departments [52]	Carcenogenic [51], Damage to liver, kidney and central nervous system, allergies, nausea, fatigue, emesis, dizziness [39,53,54], formaldehyde and benzene have been identified carcinogen [55,56,57,39]	Using low emitting products, Avoiding air fresheners, exhausting [58] Activated carbon filter [59,60]
Carbon monoxide (CO)	Parking areas, Outdoor air exchange [61].	nausea, vomiting, chest pain, and confusion neurological problems [43,55,62,61]	Fe2O3 nanoparticles may oxidize carbon monoxide [63]
CO_2_	Dry ice for preserving the cadaver [64], occupants	High concentrations leads to an increased respiratory rate, tachycardia, cardiac arrhythmias and impaired consciousness. More than 10 %, may cause convulsions, coma and death [39,55,65]	Ca-based CO_2_ Sorbents [66,67] CO_2_-based ventilation control [68]
Lead (Pb)	Hospital waste, Contaminated dust from outside [69]	Dangerous to children’s brains and nervous systems; exposure can disturb cardiac autonomic function as well [39,70]	Adsorption by Activated carbon [71].
Anesthetic gaseous agents	Ambulatory operating room, and the burn unit, postanesthesia care units. leakage into the atmosphere due to ventilator circuit connections, exhaust valves, defects in plastic insufflation balloons, or ventilator connectors [72,73].	Reproductive effects, spontaneous abortion, teratogenicity [74,75,72,73]	Photocatalytic oxidation can reduce isoflurane (Byproducts may release) [76]. Activated carbons, zeolites, metal-organic frameworks, aerogels, carbon nanotubes and activated alumina [77]
Antimicrobials biocides, sterilants, insecticides, herbicides, and fungicides)	Stored containers, disinfectants in hospitals [78]	Risk of cancers, Irritation to eye, nose and throat, damage to central nervous system, liver and kidney [39,79].	Certified cleaning agents, Rinsing of surfaces when overuse, Indoor lights to degrade insecticides [80]. TiO_2_ photocatalysis for gaseous pesticides [81].
Infectious respiratory aerosols, respiratory aerosols <5 μm Particulate Matters (PM _2.5_)	poorly ventilated spaces containing infectious individuals, medical wards polluted outdoor air exchange [82]	Multiple and Immense effect on human health, respiratory disease Premature death, particularly cardiovascular diseases, endocrine malfunction, metabolic diseases [39,83].	Portable air cleaner with HEPA filter [84,85]
SARS-CoV-2 droplets	Droplets from infected patients/staff’s mouth or nose cough, sneeze, speak, sing or breathe. Through aerosols Environmental surfaces [86]	COVID-19 Infection associated illness	Portable Air Cleaners and Combining ventilation [[87], [88], [89]]
Polycyclic aromatic hydrocarbons	Parking vehicles near hospitals [90]	Mutagenic, Carcinogenic Naphthalene lesions, Tumors in the upper respiratory tract, hemolytic Anemia [55,91]	Air filtration [92], zeolite filter [93]
Radon (naturally occurring radioactive gas)	In hospitals in basement rooms [94]	Carcinogen [95,96,97,94]	Activated charcoal [98] Appropriate intermittent ventilation [99]
Ozone (O_3_)	Possible byproducts of UV sterilization	Toxic. Ozone can also exacerbate chronic respiratory diseases such as asthma [43,55,96].	Activated carbon-based filters, catalytic decomposition [100]

In addition, bioaerosol composition and distribution in hospitals is complex. PM_2.5_ bioaerosols sampling showed *Vibrio metschnikovii*, *Staphylococcus epidermidis*, *Staphylococcus haemolyticus*, *Fusarium pseudensiforme*, and *Aspergillus ruber are dominant species in hospitals* [101]*.*

These contaminates showed spatial variation in the hospital and influenced by temperature, relative humidity, ventilation system, and daily activities. Studies showed that total bacterial load may reach 134.3 CFU/m^3^ across the intensive care units (ICUs) [102]. Multi-antibiotics resistance methicillin-resistant *Staphylococcus aureus* (MRSA) concentration was found 95 CFU/m^3^ in pediatric wards [103].

**Fig. 2:**
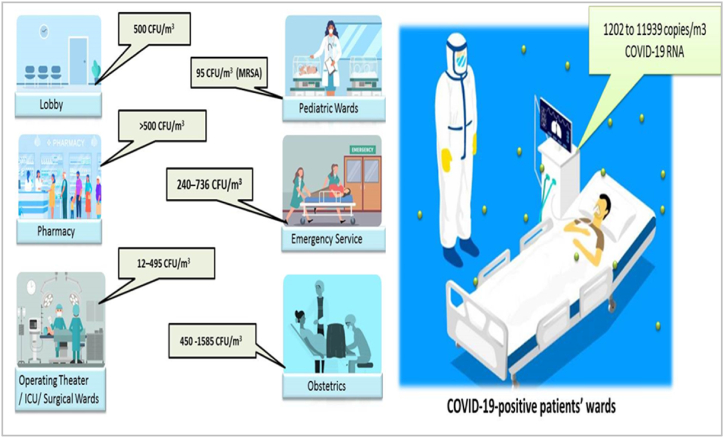
Variation in microbial concentration in different wards.

Several studies observed that bacterial concentrations may exceeded WHO guidelines in obstetrics, pediatric, and surgical wards range 450–1585 CFU/m^3^, Fig. 2 [104].

**Fig. 3:**
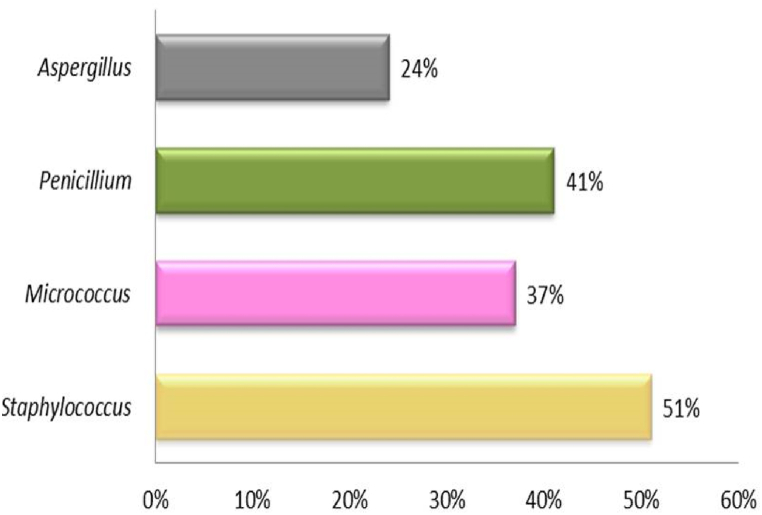
Most Frequent bacterial species in Hospital atmosphere.

A study in Singapore’s local hospital showed airborne bacteria such as normal skin microbiota, opportunistic *Acinetobacter*, and *Flavobacterium* spp 500 CFU/m^3^ in the air-conditioned lobby and pharmacy. However, in restricted wards, the maximum concentration of the same bacteria was 325 CFU/m^3^. Indoor levels of airborne bacteria were significantly correlated to relative humidity [105]. Similarly in a Portuguese hospital airborne microbial count exceeded the conformity criteria as the highest bacterial counts were 240–736 CFU/m^3^ (BC) and 27–933 CFU/m^3^ fungal load (FL) observed in emergency service sites. While Bacterial concentrations in the surgical wards range 99–495 CFU/m^3^ and the operating theater 12–170 CFU/m^3^ under recommended criteria while fungal levels were below 1 CFU/m^3^ in the operating theater, and range 1–32 CFU/m^3^ in the surgical wards. Most frequent (88 %) phenotype was Gram-positive cocci in all indoor environments which accounts *Staphylococcus* (51 %) and *Micrococcus* (37 %) and fungal prevalent genera were *Penicillium* (41 %) and *Aspergillus* (24 %), Fig. 3 [106].

**Fig. 4:**
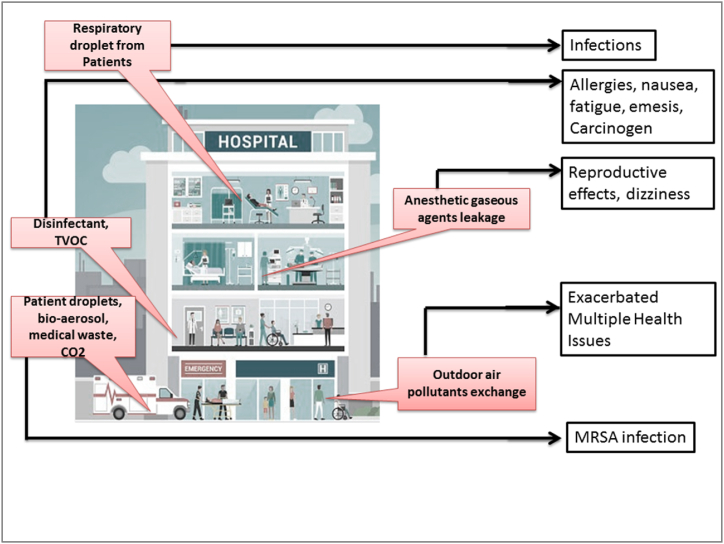
Types and sources of air pollutants in the healthcare system.

Studies showed an average 5–10 % of in-patients in hospital can be infected with nosocomial infection, mostly in intensive care and surgical units. Frequently observed nosocomial infections categories includes bacteraemia, catheter-associated urinary tract infection, gastrointestinal infection, nosocomial pneumonia and surgical wound infection [107].

However airborne respiratory viruses infections are also not less common. The load and dispersion of parainfluenza virus, respiratory syncytial virus, rhinovirus, and adenovirus were associated with the number of infected patients in wards. Air viral load is significantly higher (1.02 × 104 copies/mL) in 2-patient rooms compared to singly isolated patients (1.58 × 103 copies/mL) [108].

Airborne microbe’s frequency may be highest in respiratory patients' clinics and ICU units. In addition, the presence of some pathogens may be correlated. A noticeable correlation was found between SARS-CoV-2 concentration and the presence of airborne bacteria [109].Dissemination of airborne pathogens could be associated with several factors. A study found that serious antibiotic-resistant pathogens such as *Clostridioides difficile* can be intra-associated with hospital air, floor, and hospitalized patients [110].

Aerosol samples from different COVID-19-positive patients’ wards, rooms in long-term care homes experiencing outbreaks, and ICU rooms showed 1202 to 11939 copies/m3 of viral RNA in air, low concentrations of viral RNA in well-ventilated spaces, Fig. 2 [111].

Removal of hazardous airborne contaminates has been an important practice in healthcare settings [112]. Disinfection and air cleaning preventive measures had remarkable effectiveness to reduce HAI was observed during novel SARS-CoV-2 spread [113].

However, extensively used surface disinfectants/sterilants products such as bleach, glutaraldehyde, formaldehyde, hydrogen peroxide, and enzymatic cleaners have been proven hazardous to occupant’s health and environment. Several studies suggest that cleaning agents are associated with work-related asthma and skin irritations among hospital workers [114,115].

Hospitals may suffer from the overuse of cleaning agents and hazardous waste production, the largest contributor to environmental degradation. Healthcare waste is the fourth biggest contributor to mercury presence in the environment. Large countries like the United States produce around 6700 tons of waste daily [116]. So, cleaning and waste management have been routinely monitored aspect in hospitals in many countries [117].

Bioaerosol or microbial contaminants are often estimated in for of CFU/m^3^ to predict the overall indoor air hygiene in hospital wards. Indoor bacterial count of 9.6 × 102 cfu/m^3^, may increase the risk of respiratory infections development compared to the recommended cfu (≤500 cfu/m^3^) level by American Industrial Hygiene Association [118,2].

## 4 Routine disinfection and environmental cleaning in healthcare facilities

Environmental cleaning is crucial to prevent microbial contaminants in healthcare facilities. Common surfaces like floors, bathroom facilities, bed rails, furniture in patient rooms, and medical equipment could be reservoirs of highly contagious pathogens including multiple species of *Acinetobacter*, *Clostridium difficile*, methicillin-resistant *Staphylococcus aureus* (MRSA), and vancomycin-resistant *enterococci* (VRE). Environmental cleaning is a broad term that involves various disinfection and monitoring methods. Multiple cleaning agents and disinfection technologies are being used in health sectors.Commonly used surface disinfectants are sodium hypochlorite, quaternary ammonium compounds, peracetic acid, and liquid hydrogen peroxide. Methods like Ultraviolet light (UV-C) or fogging with hydrogen peroxide vapor for disinfection when patient rooms are empty [119]. Cold atmospheric pressure plasma and electrolyzed water (hypochlorous acid) are also being used for disinfection [120]. Other cleaning options such as self-cleaning or disinfecting floor tiles are also being considered in hospitals. Coating with several germicidal materials such as photocatalytic TiO₂ nanoparticles has shown interesting applications in the healthcare environment [121]. However, disinfectant selection should be according to occupational safety and environmental compatibility. For instance, some reusable medical equipment such as endoscopic devices can be damaged by peroxyacetic acid (PAA) solution [122].

### 4.1 Monitoring and risk assessment of biological contaminants

Regular air and surface monitoring are necessary to detect potential contaminants in healthcare settings. However, evidence-based risk analysis protocols are limited. Healthcare facilities may operate patient, pathogen, and surface risk assessment. Microbiological sampling is the most common practice to monitor the cleanliness in hospitals but delayed results cause hindrance. Advanced methods such as adenosine triphosphate (ATP) bioluminescence assays or ATP meters are gaining popularity in the hospital environment due to easy operation and quick results, ATP is an important energy source in every living contaminates but its routine use, remains controversial [123].

Significant variation can be seen in surface and air sampling methods. Basic surface sampling involves direct sampling (contact plates, dipslides, Petrifilms) and indirect sampling (swabs, sponges, wipes). Collected samples further culture on suitable media and investigates. While for specific pathogens specifically designed swabs and media are used. For instance, macrofoam swabs and tryptone soya broth media are used to collect and grow MRSA respectively, and a sponge swab and Brazier's CCEY agar are used for *C. difficile*. For viruses DNA or RNA-based methods are used. In addition, Matrix-assisted laser desorption ionization-time of flight (MALDI-TOF) mass spectrometry technique, PCR, qPCR, and Multiplex PCR can be used for accurate results [124].

For bioaerosol microbiological sampling, passive air monitoring and active air monitoring are two commonly used methods. In passive monitoring, standard Petri dishes with suitable media are exposed to the air, (also known as settle plates) in the investigation area for a few hours, that’s allows biological particle sedimentation in Petri dishes. These plates are incubated and results are expressed in CFU (Colony Forming Units)/plate/time or CFU/m^2^/hour. This method is also useful for valid risk assessment of harmful parts of the airborne pathogens in surgical wards. Active air monitoring involves a microbiological air sampler that allows a specific known volume of air through or over a particle collection object which can be a solid culture media plate or liquid or a nitrocellulose membrane filter. These media or filters are further processed for the microbial load quantity, measured in CFU/m^3^ of air. This method is preferred in controlled environments such as operating theatres where airborne pathogens concentration is supposed to be low [125].

Studies suggest that an air sampler can be an effective tool to collect a variety of airborne particles. Specifically designed air samplers including vacuum cleaner-based air samplers have been successfully used for SARS-CoV-2 sampling [[126], [127], [128]].

For chemical pollutants such as total volatile organic compounds (TVOC), nitrogen dioxide, particulate matter (PM2.5), and CO2. Sensors-based real-time air quality monitors were found useful [129,130].

### 4.2 Sanitary standards for biological contaminants

Bio-aerosols contain a range of tiny particles airborne particles (ranging from 0.001 to 100 μm) of bacteria, endotoxins, fungi, mycotoxins, and allergens. However, health risk-based standards for bio-aerosols are limited and have variations as there are no uniform international standards to determine acceptable bacterial loads in indoor air. A group of WHO experts suggest that total microbial load should not exceed 1000 CFU/m^3^ in common indoor environments. Whereas other researchers suggest 800 CFU/m^3^ maximum upper limit for ubiquitous bacterial aerosol. For fungi 150 CFU/m^3^ of mixed species may be acceptable but 50 CFU/m^3^ for single species of fungi warrants immediate examination [131,132].

The European Commission's sanitary standards for bacterial and fungal load in non-industrial premises have been considered helpful in several airborne pathogens investigations in healthcare facilities, Table 2 [133,134].

**Table 2:**
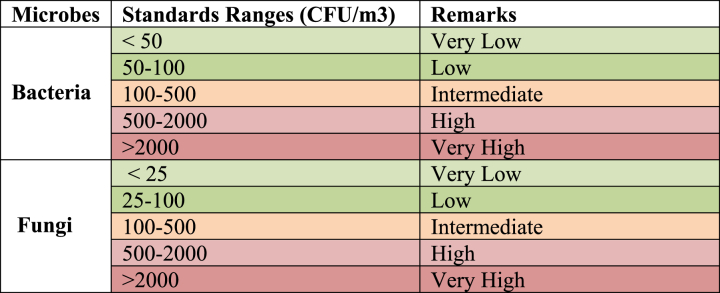
The European Commission's sanitary standards for biological contaminants in non-industrial premises.

In addition, for individual safety, medical face masks and respirators may provide significant personal protection against pathogens containing bio-aerosols including viruses [135]. Occupational Safety and Health Administration (OSHA) provided specific recommendations to protect high-risk workers in healthcare from infections like COVID-19 which involves the principles of ventilation, physical distancing, and face coverings. Medical masks must comply the international standards such as Food and Drug Administration (FDA) or American Society for Testing and Materials (ASTM) standards to ensure that the face mask or similar device has an acceptable level of filtration, biocompatibility, and breathing resistance [136].

## 5 Airborne droplets transmission control in different wards

Highly transmissible Pathogens control could be challenging in healthcare settings due to several constraints. For instance, to check the air hygiene Colony Forming Units (CFU) count found useful proposed standards to evaluate bacterial and fungal contamination. In most cases, a total aerobic colony forming units count of <2.5 or 5 CFU per cm^2^ and <1 CFU/cm^2^ of *Staphylococcus aureus*, *Enterococcus* species, etc. is recommended. However, no such standards apply to viral aerosol and in the emergency medical services [137].

According to the Healthcare Infection Control Practices Advisory Committee (HICPAC), the four classes of infection control: Standard Precautions, Droplet Precautions, Contact precautions, and airborne precautions are recommended to control airborne pathogens transmission.

**Standard Precautions** involve common hygiene practices to avoid contact with blood, body fluids, all secretions, nonintact skin, and mucous membranes. Infectious patients must be placed in a private room. **Droplet precautions** involve the prevention of coughing and sneezing droplets exposure from infected ones. Since droplets usually travel only 3 feet in the air face mask, private rooms are suggested to avoid exposure. **Contact precautions** involve skin-to-skin contact. Gloves and sterile patient care equipment can be dedicated to a single uses. **Airborne precaution** requires careful air handling and ventilation. Infected Patient's rooms should have negative air pressure and 6–12 ACH per hour and no air recirculated to other areas without significant filtration. Personal respiratory protection such as N95 respirators is suggested when entering the room [138].

### 5.1 Ventilation strategies

Increased ventilation mechanical or natural can reduce airborne transmission effectively. Appropriate air distribution and airflow patterns are important engineering intervening approaches against airborne droplets including respiratory infectious diseases COVID-19. Ventilation indices of air change effectiveness (ACE) or air change per hour (ACH) are the most commonly used parameters to evaluate indoor air recirculation [139,140,141].

Hospital ventilation conditions may influenced by different climate conditions and indoor designs. Usually, four types of hospital ward designs are preferred worldwide: bay wards, nightingale wards, racetrack wards, and hub and spoke units. The nightingale wards may cater to up to 30 adults or 24 children and is still widely used in many developing countries. Indoor conditions in operating rooms, isolation rooms, and nursing wards have specific HVAC guidelines; commonly recommended temperature, relative humidity, ACH, pressurization range, and worldwide variation thereof are given in Table 3 [142].

**Table 3:** Worldwide HVAC guidelines for different wards in hospitals.

Types of Wards	Temperature C	Relative Humidity	Recommended Minimum ACH (Indoor)	Suggested Air Pressure	References
Patients Room	21–23	30–60 %	6	–	[142,143]
Pediatric& obstetrician wards	22–26	30–60 %	6	–	[143]
Surgery Room	17–27	30–55 %	15–25	Positive/neutral	[143,144]
General inpatients area and corridor	≤24	30–60 %	4	–	[143]
Isolation wards with highly infectious patients like COVID-19	–	–	12–15	Negative	[144]
ICU	21–24 °C (Australia) 16–25 °C (India) 21–24 °C (UAE) 18–25 °C (UK) 21–24 °C (USA)	**30**–**60 %** (Australia, UAE, USA) 40–60 % (UK)	**6** (Australia, India, UAE, USA) **10** (UK)	Positive (Australia) Neutral (Germany) Neutral (India) Positive (UAE) Positive/Neutral(UK) Neutral(USA)	[144]

The ventilation efficiency can be increased with proper filtration of natural fresh air and by reducing the occupancy in patients' wards and concentration of exhaled aerosols [145,146].

Recirculated air should be filtered in wards, especially in isolation wards with highly infectious patients.

To minimize the risk of airborne contaminations spread medical facilities must require MERV 14 to 16 rating filters. These filters with MERV ≥17 are known as high-efficiency particulate air (HEPA) filters. A typical MERV 17 rating HEPA filter has an efficiency of 99.97 % against 0.3 μm size particles. However, in highly hygienic body implant areas MERV rating can be 20 with a filtration efficiency of 99.999 %. Microbial count expected below 0.1 CFU/m3 where HEPA filtration is employed. HEPA filters need regular monitoring every 6 months [144].

Multi-bed spaces like Nightingale wards are common in many countries. Contamination-free proper ventilation in large multi-bed wards could be challenging due to multiple openings. High ventilation rates are achievable in naturally ventilated hospitals. A nightingale ward showed that external wind 1–4 m/s speeds may lead to indoor ventilation rates as recommended for general wards (3.4–6.5 ACH). Extractor fans can also play a key role in maintaining air quality. However, a hybrid ventilation system with natural and mechanical ventilation may deliver the best year-round solution [147].

**Fig. 5:**
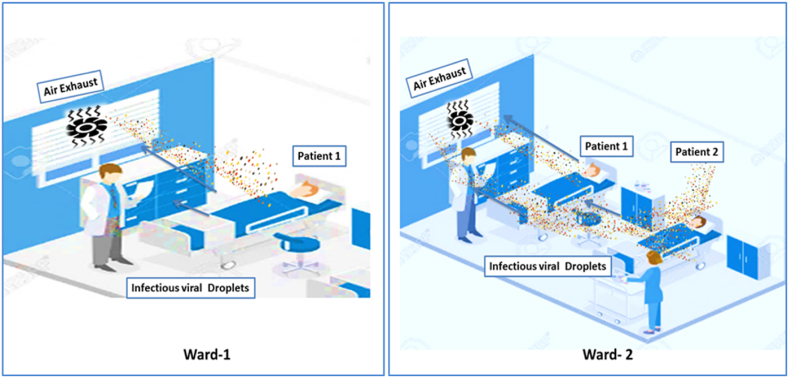
Infected patients' positions and the risk of cross-contamination: Ward-1: Patient 1 position near air exhaust may reduce the risk of cross-contamination as infectious droplets eliminate swiftly. Ward-2: Patient 2's position facilitates longer traveling distances of infectious droplets, so the risk of cross-contamination and surface contamination may increase.

The location of an infected patient in hospital wards may affect infections like MERS-CoV, SARS-CoV, and H1N1 influenza virus spread risks to other occupants in the same hospital. It is observed that patients’ position near the corridor can exhaust the viral droplets into the corridor immediately, Fig. 5. Contrary infected patient's position in the inner part of the ward allows the virus particles to deposit on wall surfaces or other patients. Usually, 9 ACH and the installation of ultraviolet germicidal irradiation (UVGI) lamps can minimize the risk of highly infectious viral droplets [148].

Toilets in the wards can also be the hub of virus transmission so must not be neglected strict infection control precautions including ventilation by opening any windows or by propping extractor fans can be increased. The movement of near infectious patient’s wards should be reduced as far as possible [149].

## 6 Air Filtration Strategies

The importance of ventilation in indoor pollutant dilution is discussed in the 4th section. Contamination-free ventilation systems are expected in hospitals. Studies show negative pressure, adaptive wall-based attachment ventilation with HEPA filters, adsorption filters, and UV radiation could be beneficial in a surge of COVID-19-like infection control. However, the concern of associated byproducts such as microplastics, and residues of degrading filters also increased [150,151].

### 6.1 Negative pressure wards significance in the surge of COVID-19-like diseases

**Fig. 6:**
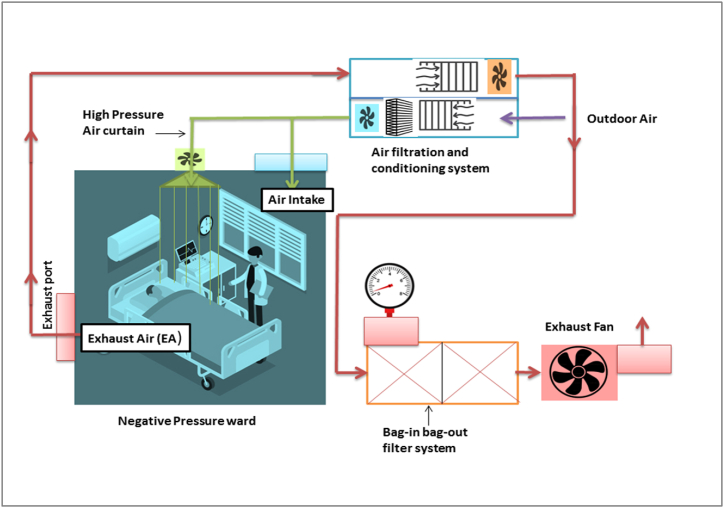
Negative Pressure ward with high pressure air curtain and filtered air supply.

**Fig. 7:**
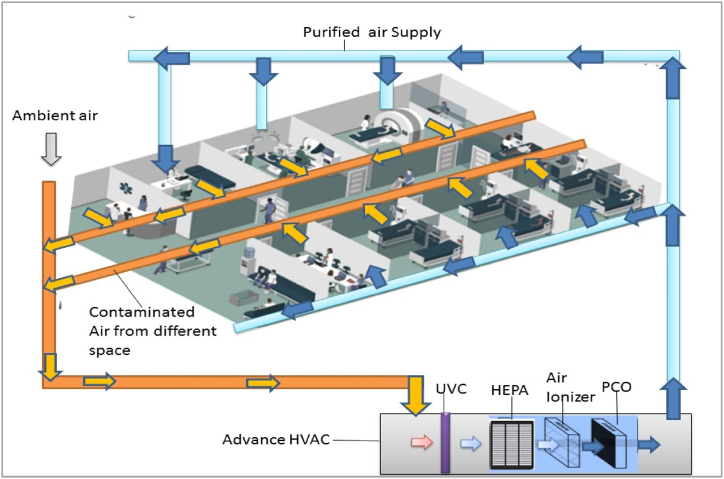
Air cleaning Interventions combined with central HVAC system in Hospital (HEPA grade 14).

In 2020, as many as 8000 medical personnel were infected 63 and physicians died from COVID-19 in Italy. The major cause of frontline medical system collapse is enclosed medical environments between patients and medical staff and an insufficient number of negative pressure isolation wards to admitting infected patients. Indoor air quality in medical spaces can be affected by external air pollution such as waste gasses, diesel exhaust, hydrocarbon particulate matter and ozone, and other pollutants directly generated indoors such as volatile organic compounds (VOCs) from indoor interior decoration, furnishings and carbon dioxide (CO_2_) exhaled by humans. CO_2_ concentration in indoor environments plays a key role in indicating indicators of indoor air quality; it is recommended that CO_2_ concentration in hospitals should be less than 600 ppm. Negative pressure isolation wards contain ventilation systems that are generally well planned, but the issue of nosocomial and air cross-contamination is primarily due to the factor of indoor air quality (IAQ). Negative pressure isolation wards usually contain well-planned ventilation systems to keep indoor environments healthy but are associated with emissions that create heat and air pollution in the environment. Negative pressure should be up to 8∼12 Pa in the wards and hallways to prevent contaminated air from entering other spaces. It has been tested that increasing the air change rate 54.5 and 40 times/hour and introducing fresh air to maintain CO_2_ concentration below 600 ppm can increase the efficiency of traditional negative pressure isolation wards. In addition, A velocity of 0.5 m/s for an air-jet curtain in negative pressure ward can also decrease the contamination and exposure of aerosol particles from the coughing patient, Fig. 6 [152,153] (see Fig. 7).

SARS-CoV-2 variants such as BF.7, BA.2, BA.2.75, BA.2.75.2, BQ, XBB, etc. concern have been continuous since the pandemic erupted. Most of these variants could accelerate the emergency admission in hospitals and posed substantial pressure in health care systems. New variants may acquire exclusive characteristics such as elevated immune evasion, additional spike mutations, and higher transmissibility. Amid the continuous episode of new variants recent variant JN.1 burgled the alarm once again as detected in 41 countries. Major Prevalence from 1.0 % to 9.9 % for Canada, 10.9 %–45.5 % for France, from 2.1 % to 19.9 % for the United States of America, from 1.4 % to 72.7 % for Singapore, from 1.8 % to 20.4 % for the United Kingdom, and from 1.8 % to 22.9 % for Sweden is observed. This variant is spreading rapidly and is anticipated to cause a surge in SARS-CoV-2 cases and overlap with other viral and bacterial infections, especially in countries entering the winter season [154,155]. So it is crucial to review and prepare hospital isolation wards with sufficient and more efficient negative pressure wards for unpredicted infection outbreaks [156].

### 6.2 Portable air purifiers and air filters

Recent findings show that air cleaners or mechanical ventilation equipped with a combination of pre-filters, High-Efficiency Particulate Air (HEPA) filters, and four-cylinder gas filter cartridges can reduces formaldehyde, odors, and TVOC, PM_2.5_ and improve indoor air quality in clinics [157,158]. Air purifiers used with HVAC systems in hospitals may enhance the indoor hygiene by reducing air contaminants [159]. Portable air purifiers can be a supplementary measure to reduce the exposure risk of multiple airborne indoor air pollutants in hospital settings, particularly when aerosol transmission control not possible by HVAC [[160], [161], [162], [163]].

HEPA filters with the highest MERV rating, ultra-low particulate air filters (ULPA), and ultraviolet air filtration ionizers, alone or in combination showed significant impact, ranging from 12 to 99 % on indoor bioaerosol and other pollutants. Research showed HEPA filter capacity can be increased by ultraviolet radiation, with coating material such as covered with tannic acid and air ionizers [164,165,166].

Highly infectious SARS-CoV-2 viral RNA in the air of intensive care units and corridors could be difficult to remove by conventional air cleaning method [167]. Airborne pathogenic bacteria, fungi, and viruses could be around 5–34 % of total indoor air pollution. Experiments show the highest bacterial (829–4980 CFU/m^3^) and fungal levels (90–920 CFU/m^3^) in OPDs while the lowest concentration in OTs [168]. Therefore, germicidal treatment of filtered air by ionization and UV radiation is suggested [169].

### 6.3 Bipolar air ionization, ultraviolet germicidal irradiation (UVGI) and electric precipitations

**Fig. 8:**
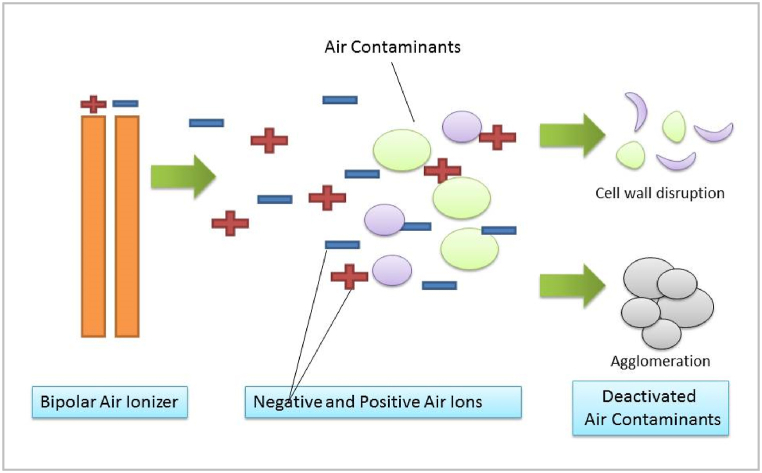
Air ions associated possible deactivation of airborne contaminates.

Air ionization has been a low-maintenance and effective technique to decrease several indoor air pollutants, especially, bipolar ionization which gain significant attention during the COVID-19 pandemic and was installed in many places including hospitals. Bipolar air ionizers produce both positive and negative ions that react with various airborne contaminants (Fig. 8) including volatile organic compounds, particulate matter, and airborne pathogens [170].

The evidence of bipolar air ionizers’ air cleaning efficacies is growing, and disinfection capacity against *E.coli*, *S. typhimurium*, and *S. epidermidis* is promising. Yet, the potential byproduct of O_3_ might be associated with some air ionization devices operation [171,172].

Ultraviolet germicidal irradiation (UVGI) or Ultraviolet C has been known for shorter wave length (100–280 nm) and highest disinfectant capacity than UV-A and UV-B rays. UVC usually absorb by pathogen nucleic acids (DNA, RNA), which halted their ability to replicate. A large number of pathogens have been found sensitive to UVC, although highly dependent on UVC dose. UVC disinfectant has shown 99.99 % germ eradication. UVC equipment has been successfully employed in hospitals wards for decontaminations [173]. Additionally, UVC and air ions combination may lead to higher inactivation against fastidious airborne pathogens [174].

Electrostatic precipitation (ESP) is an electric field-based technique that charged airborne pollutants in a gaseous medium, conventionally used in industries to manage the smoke and particulate pollutants. In the presence of an electric field charged pollutants are directed towards a metallic collection plate. However, compared to bipolar air ionization and HEPA, ESP may be considered less efficient when indoor pollution is high. Moreover, byproducts like ozone generation during corona discharge are still a challenging phenomenon [175].

## 7 Advanced HVAC system

Poorly designed HVAC may be responsible for to spread of nosocomial infections as a strong correlation is found between ventilation and air movement in buildings. Most conventional HVAC systems focus on air temperature and CO_2_. Though other parameters such as RH, negative pressure, air flow rate, laminar flow ceiling, and Local exhaust ventilation to remove anesthetic gases, special HVAC designs for OTs are recommended to enhance the HVAC performance [176].

Studies indicated that additional filtration and disinfectant amalgamation may enhance the HVAC capacity. For instance, air changes per hour (ACH), Table 1 [177], and the use of heat pipe heat exchangers with HVAC may increase the indoor contamination reduction. In addition, uses of HEPA or ULPA with central HVAC or within the room via portable air purifier, Laminar air flow at celling of emergency wards, controlled and constant relative humidity, UV light treatment of air stream, and displaced ventilation instead of mixed ventilation can enhance the indoor air quality and hygiene in hospitals [178].

## 8 Photocatalytic oxidation (PCO), plants and Trombe wall role to manage nearby outdoor air pollutants

Parking area near healthcare may be responsible to increase the exposure risk from parked vehicle emission. Photocatalytic oxidation (PCO) air cleaning technology has been considered a feasible and energy-efficient method to improve Air Quality. Photocatalysts such as TiO_2_ oxidation reactions can convert/breakdown the airborne pollutants into CO_2_ and water molecules. Photocatalytic oxidation can be useful to improve air quality in hospitals, especially to reduce hospital-borne pollutants such as anesthetic gas, and isoflurane [179]. Photocatalytic oxidation-based air cleaning systems' positive effects in hospitals and clinics are growing. Photocatalyst TiO_2_ nanoparticles and optical fibers were found effective in glutaraldehyde and Chloroform removal [180]. Another similar study also suggested that Photocatalyst TiO_2_, with plasma, and UV can remove the hazardous chloroform from hospitals [181].

Plants are the natural air cleaner and able to reduce decrease certain air pollutants by phytoremediation which involved dilution, absorption, filtration, and precipitation. Thus in certain areas, such as near hospital entrance and corridor, plants may increase the occupants comfort by reducing certain indoor air pollutants. However, indoor plants may posse potential health risks [182,183].

Substantial energy is consumed by buildings to maintain an indoor environment comfortable, especially thermal comfort. Trombe wall might be a sustainable solution to support and enhance HVAC system efficiency in certain regions [184]. The Trombe wall contains breathing wall panels that also filter air along with heat. Trombe walls facilitate a higher ventilation level for buildings associated with a large reaction area, thus improving indoor air quality by reducing energy consumption [185].

## 9 Organic pollutants abatement by passive removal materials (PRMs)

Indoor organic pollutant removal is not always possible through source control and ventilation. To overcome the issue of indoor air pollutants an energy-efficient method Passive removal materials (PRMs) have been proposed by various research. These materials efficiently remove organic pollutants without by-product formation and with minimal energy consumption [186,187].

These Materials usually are clay-based bricks, plasters, calcareous stone, activated carbon, mineral fibers or volcanic perlite, and manganese oxides. Studies showed that PRMs decomposed formaldehyde and ozone when employed as clay paint on walls, and ceiling tile panels in buildings at room temperature without mechanical intervention [[186], [188], [189], [190]].

Sorbent sinks made by activated carbon remove Volatile Organic Compounds (VOC) such as acetone and formic acid, acetic acid, toluene, and alpha-pinene from the air [[191], [192], [193]].

Nanomaterial-based biosorbents may also be used for viral droplets inactivation due to their broad-spectrum antiviral capabilities [194]. Nanomaterials may block the aerosol transmission route of the virus and deactivate viruses via the generation of ROS, physical contact, photo-thermal effects, catalytic oxidation, and metal ion release. Such nanomaterials can also be applied to building ventilation systems, air purifier filters, anti-viral fabrics, and anti-viral sprays [195,196].

However, PRM practicality and inactivation of SARS-CoV-2 in hospital wards remain the subject of extensive research.

## 10 Air cleaning Intervention in Emergency Health Vehicles

**Fig. 9:**
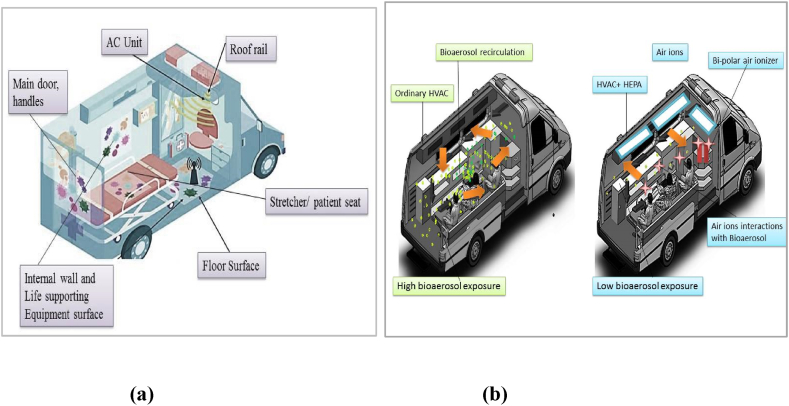
The potential reservoir of contaminants in ambulance cabin (a); air contaminants control with inbuilt HEPA and Air ionizer in cabin (Image is retrieved and modified from Zhou et al., 2022, https://www.mdpi.com/2071-1050/September%2014,%204900).

The potential health risk of in-cabin PM_2.5_ and other contaminants exposure in emergency vehicles for medical staff cannot be neglected. Emergency health vehicles or ambulances are an important part of health care facilities and a vulnerable source of many airborne microbial contaminants due to the exposure to patients’ secretions i.e. blood, excreta, respiratory droplets, medical waste etc. Ambulance-associated infections not only create a risk for patients and paramedical staff but also a significant financial burden on the entire healthcare system. Various internal parts of ambulance may be reservoir and responsible for contaminants dispersion, Fig. 9 (a). The most common difficult-to-treat ambulance-acquired infection is methicillin-resistant *Staphylococcus aureus* (MRSA) [197]. Airborne bacterial and fungal load is comparatively high when ambulances run with patients, indoor CFU can reach up to 468 ± 607 cfu/m^3^ for bacteria and 656 ± 612 cfu/m^3^ for fungi. However other air pollutants such as particulate can affect the indoor air quality of ambulances [198,199]. Disinfectants residues such as hydrogen peroxide mist can also affect the indoor air quality [200].

A recent pilot study shows that a vehicle's heating, ventilation, and air-conditioning (HVAC) system with filters with a high minimum efficiency reporting value (MERV) can reduce in-cabin pollutants, allergens, and airborne viral particles. In addition, portable air purifiers may efficiently remove PM_2.5_ by 42–74 %, less than the 15 μg/m^3^ threshold recommended by the WHO [201]. Ambulance services can be affected in highly polluted countries; a significant toxic effect of PM_2.5_ with hot and humid climate has been observed [202].

The technique such as negative pressure has been useful to check contamination spread. Negative pressure involved a fan with a high-efficiency filtration device (HEPA) to filter the air in the cabin and create low cabin pressure to minimize the highly contagious viral droplet exposure. Such filtration may minimize the viral contaminants exposure. Such filtration may minimize the viral contaminants exposure, but limitations like insufficient airflow, uncertain distribution of droplet particles, and patient coughs or sneezes during mask change may increase the risk to medical health workers [203]. A safe real-time germicidal and decontamination system could be helpful to minimize continuous exposure to biological and chemical contaminants during patient transportation. For instance, a bipolar ionization unit may be useful as an additional tool to reduce/deactivate airborne pathogens which managed to escape due to low airflow and HEPA malfunction, Fig. 9 (b).

A recent experiment showed that bipolar ionization and higher CADR of air cleaning devices were able to reduce environmental bioaerosols in public vehicle like tramps [204].Growing research is showing that bipolar air ionization may be a low-maintenance and effective air-cleaning intervention to abate a variety of biological (including *Clostridioides difficile*, *Klebsiella pneumoniae,* MRSA, carbapenemase-producing *K. pneumoniae*, and multi-drug-resistant *S. aureus*) and chemical contaminants [[205], [206], [207]]. Studies are adding evidence that appropriate ventilation with air-cleaning devices could lower the chance of occupants' associated airborne pathogens and tracer gas exposure [208].

## 11 Conclusion

The amalgamation of recent air cleaning technologies including increased ventilation, modified air filters with the highest MERV, bipolar air ionization, Passive Removal Materials, irradiation, negative air pressure, and specified HVAC standards for different wards may help to eliminate the risk of airborne contaminates and other indoor air pollutants in health care facilities. These strategies proposed a potential solution for unpredicted airborne COVID-19-like outbreaks. However, the practicality of these techniques may be subjected to further extensive research.

## Funding

The current work was assisted financially to the Dean of Science and Research at the 10.13039/501100007446King Khalid University via the large group project under the grant number RGP.2/409/45.

## CRediT authorship contribution statement

**Nishant Gupta:** Writing – original draft, Project administration, Methodology, Data curation, Conceptualization. **N.S. Abd EL-Gawaad:** Writing – review & editing, Resources, Investigation, Formal analysis. **L.O. Mallasiy:** Writing – review & editing, Visualization, Validation, Supervision.

## Declaration of competing interest

Authors declare no conflict of interest.
